# Radiographic characterisation of spinal curvature development in farmed New Zealand Chinook salmon *Oncorhynchus tshawytscha* throughout seawater production

**DOI:** 10.1038/s41598-020-77121-y

**Published:** 2020-11-18

**Authors:** B. A. Lovett, E. C. Firth, I. D. Tuck, J. E. Symonds, S. P. Walker, M. R. Perrott, P. S. Davie, J. S. Munday, M. A. Preece, N. A. Herbert

**Affiliations:** 1 Institute of Marine Science, University of Auckland, Auckland, 1010 New Zealand; 2 Liggins Institute, University of Auckland, Auckland, 1023 New Zealand; 3 National Institute of Water and Atmospheric Research, Auckland, 1010 New Zealand; 4 Cawthron Institute, Nelson, 7010 New Zealand; 5 School of Veterinary Science, Massey University, Palmerston North, 4474 New Zealand; 6 The New Zealand King Salmon Company Ltd., Picton, 7220 New Zealand

**Keywords:** Animal physiology, Zoology, Bone

## Abstract

Spinal anomalies are a recognised source of downgrading in finfish aquaculture, but identifying their cause(s) is difficult and often requires extensive knowledge of the underlying pathology. Late-onset spinal curvatures (lordosis, kyphosis, scoliosis) can affect up to 40% of farmed New Zealand Chinook (king) salmon (*Oncorhynchus tshawytscha*) at harvest, but little is known about their pathogenesis. Curvature development was radiographically documented in two related cohorts of commercially-farmed Chinook salmon throughout seawater production to determine (1) the timing of radiographic onset and relationships between (2) the curvature types, (3) the spinal regions in which they develop and (4) their associations with co-existing vertebral body anomalies (vertebral compression, fusion and vertical shift). Onset of curvature varied between individuals, but initially occurred eight months post-seawater transfer. There were strong associations between the three curvature types and the four recognised spinal regions: lordosis was predominantly observed in regions (R)1 and R3, kyphosis in R2 and R4, manifesting as a distinct pattern of alternating lordosis and kyphosis from head to tail. This was subsequently accompanied by scoliosis, which primarily manifested in spinal regions R2 and R3, where most of the anaerobic musculature is concentrated. Co-existing vertebral body anomalies, of which vertebral compression and vertical shift were most common, appeared to arise either independent of curvature development or as secondary effects. Our results suggest that spinal curvature in farmed New Zealand Chinook salmon constitutes a late-onset, rapidly-developing lordosis–kyphosis–scoliosis (LKS) curvature complex with a possible neuromuscular origin.

## Introduction

Spinal anomalies, defined as any deviation of the spinal (vertebral) column from the average or norm^1,2^, occur frequently in wild and farmed finfish and can significantly impact the reputation and production of aquaculture operations^3–8^. Due to their distorted shape, severely affected individuals cannot be sold as premium products, are more difficult and costly to process and raise ethical concerns^1–3,5,8–13^. Minimising the prevalence of spinal anomalies therefore constitutes an important aquaculture management issue. However, identifying their cause(s) is difficult. For example, a single factor can produce several different anomaly types, while several factors can produce the same type^14^. Similar types can also exist as early, intermediate or final stages of the pathogenesis of different conditions^15^. In Atlantic salmon (*Salmo salar*), loss of intervertebral space can be considered to represent both an advanced stage of vertebral compression and an early stage of vertebral fusion^15–17^. Additionally, as spinal anomalies are known to arise from the interaction of multiple risk factors, whose individual effects are difficult to disentangle in commercial environments, attributing a specific factor to a particular anomaly can be challenging^2,14,15,18^. The processes by which spinal anomalies develop also often vary between species, as well as between populations of the same species reared under different conditions^1^. So while existing research may provide valuable clues, elucidating the pathology of spinal anomalies of unknown cause, and subsequently identifying the responsible risk factors, requires independent investigation.

In Atlantic salmon, alterations of the vertebral bodies (i.e. compressions and fusions) are the predominating spinal anomalies^4,15–17,19^. Deviations of the entire vertebral column, as in spinal curvatures, are comparatively rare. However, in Chinook (king) salmon (*Oncorhynchus tshawytscha*) farmed in New Zealand (NZ), spinal curvatures in the form of lordosis (downwards), kyphosis (upwards) and scoliosis (lateral) comprise the majority of spinal aberrations^20–23^, and can affect up to 40% of individuals at harvest^24^. The curvatures are known to appear late in production, in the last nine months of the seawater cycle^23^, but their exact point of onset, and thus the critical seawater period(s) for their development, remains to be identified. Spinal curvatures are also known to develop in the early hatchery stages of Chinook salmon production^25^, but these anomalies are rare in smolts^24^, suggesting that curvatures which appear later during seawater production may arise by different mechanisms. Although termed “LKS” (lordosis–kyphosis–scoliosis), the three curvature types can develop in isolation or in various combinations within an individual. It therefore remains to be established whether the curvature types are, in fact, related, or at least one arises via a separate aetiology. In some cases the curvatures are accompanied by concurrent vertebral body anomalies, but it is not known which is the primary anomaly^23^. While risk factors such as family structure^26–28^, high rearing temperatures^29,30^, vitamin C deficiency^31^, swim bladder malfunction^32–35^, high intensity swimming^34,36–38^, heavy metal toxicity^39^ and infectious agents^40,41^ have been linked to curvature development in other species of farmed finfish, the cause in Chinook salmon is presently unknown. Evidence from previous studies of farmed Chinook salmon suggests a neuromuscular origin for the curvatures^21,23^: both Perrott et al*.*^23^ and Munday et al*.*^22^ observed curvature in absence of concurrent pathological changes to the vertebral bodies in harvest-size individuals, which is considered indicative of a primary neuromuscular, rather than bone-related, deformity process^15^. In particular, unilateral fibrosis of the peri-vertebral soft tissues was found to be strongly associated with individuals with curvature^22^. However, as these investigations were predominantly focused on the end stages of development. Much about their pathogenesis remains uncertain, impeding efforts to identify the underlying causative factors.

The principal aim of this investigation was to describe the development of spinal curvatures in farmed NZ Chinook salmon throughout seawater production. In particular, we sought to document several fundamental aspects of their pathogenesis: (1) the timing of radiographic curvature onset, (2) the associations between the three curvature types and (3) the spinal regions in which they occur, and (4) the relationships of the curvatures with vertebral body anomalies, namely vertebral compressions, fusions and vertical shifts. To do this, we radiographically assessed two related cohorts of commercially-farmed Chinook salmon smolts, one tagged and one untagged, at several time-points between seawater transfer and harvest.

## Results

### Study I

#### Mortality

Mortality for the pre-grading period (M0–M6) was 5.7% (n = 45,03). Less than 1% of the population died during each of the periods from M0–M5, and 3.5% (n = 2,720) died between M5 and M6. Early runts accounted for most (69.8%) of the mortalities, with deaths of no apparent cause (i.e. clean carcasses) and late runts (7.4%) comprising the next two significant causes (9.2% and 7.4% respectively).

Post-grading (M6–M13), 31.8% (n = 8,252) of the large-grade population (n = 25,990) died. Most of this mortality occurred across the M8–M11 period, during which 25.8% (n = 6,715) of the population died. A further 0.7% (n = 190) and 5.2% (n = 1,347) died during the respective M6–M8 and M11–M13 periods. Late runts, lesions and bloat were the most significant causes, accounting for 39.4%, 26.5% and 13.9% of mortalities respectively.

Spinal anomalies were the reported cause of just 0.7% (n = 30) and 1.0% (n = 84) of mortalities in the respective pre- and post-grading periods, most of which (59.6%, n = 68) occurred in M8–M11. The M11–M13 and M0–M1 periods accounted for a further 13.2% (n = 15) and 9.6% (n = 11) of this mortality, while the remaining assessment periods comprised 0.9–7.0%.

#### Onset, prevalence and severity of spinal curvature

During this study, radiographic spinal curvature (rSC) was recorded in a total of 284 individuals, half of which were also visually affected (Table 1). Though just 3.2% of individuals sampled during month (M)1–M8 inclusive had rSC, prevalence in M2, 4 and 5 was significantly lower than M1 (χ^2^ = 6.65–13.00, p < 0.05). Significant increases in prevalence were also observed between M5 and M8 (χ^2^ = 17.38, p < 0.01), during which time the pens were graded and assessments were switched to the large-grade population only, and M8 and M11 (χ^2^ = 62.61, p < 0.01). Although higher, rSC prevalence in M13 was not significantly different from M11 (χ^2^ = 2.29, p = 0.08). The prevalence of visually-detectable spinal curvature increased similarly to that of rSC across the study (Table 1) and was positively associated with summed curvature severity (*r*_PB_ = 0.85, p < 0.01). Average curvature severity increased with time, with the greatest increase in severity occurring between M8 and M11.

**Table 1:** Prevalence of visual and radiographic spinal curvature in Study I population at assessments 1, 2, 4, 5, 8, 11 and 13 (harvest) months post-seawater transfer. *L *lordosis, *K *kyphosis, *S *scoliosis. Values in brackets represent numbers of affected individuals. n = 1,961. *Indicates where prevalence at a given assessment was significantly different (p < 0.05) than the immediately preceding assessment.

Month	n	Spinal curvature prevalence		L	K	S
		Visual	Radiographic			
1	205	0.0% (0)	5.4% (11)	2.0% (4)	2.4% (5)	1.0% (2)
2	294	0.0% (0)	0.3% (1)*	0.0% (0)	0.3% (1)	0.0% (0)
4	288	0.0% (0)	0.7% (2)	0.7% (2)	0.0% (0)	0.0% (0)
5	294	0.3% (1)	1.4% (4)	0.3% (1)	1.0% (3)	0.0% (0)
8	277	0.7% (2)	9.0% (25)*	5.8% (16)	5.1% (14)	0.0% (0)
11	303	17.8% (54)*	37.0% (112)*	26.4% (80)	29.0% (88)	12.2% (37)
13	300	28.3% (85)*	43.0% (129)	34.0% (102)	38.0% (114)	25.3% (76)
Total	1,961	7.2% (142)	14.5% (284)	72.2% (205)	79.2% (225)	40.5% (115)

Prevalence of each curvature type increased with time and peaked in M13 (Table 1). At five of the seven assessments, kyphosis (K) was the most common curvature type, and was found in 79.2% of the total 284 individuals diagnosed with rSC throughout the study period. Lordosis (L) occurred at a slightly lower rate, affecting 72.2% of the overall rSC population, while scoliosis (S) was by far the least prevalent, affecting less than half (40.5%) of rSC individuals.

Average BW of individuals with rSC was consistently lower than unaffected individuals but only significant at M13 (harvest) (*t* = 2.84, p = 0.01) (Supplementary Table 1). Affected individuals also had higher prevalences of spots, lesions and fin split/rot but lower prevalences of eye anomalies and scale loss (Supplementary Table 2).

#### Relationships between curvature types

Nearly half (42.3%) of the individuals diagnosed with rSC had only one curvature type, typically either kyphosis or lordosis (Table 2). Scoliosis was rarely observed alone. Co-existence of curvature types was first observed in M8 and increased thereafter. At M13, 54.3% of affected individuals had all three types. Associations between the curvature types were significant (χ^2^_ΜΗ_ = 20.02, p < 0.01). Most individuals affected by two types had lordosis and kyphosis (L + K); L + S was never observed and K + S was rare. L + K + S was the most prevalent curvature combination.

**Table 2:** Prevalence of radiographic spinal curvature (rSC) combinations in Study I population at assessments 1, 2, 4, 5, 8, 11 and 13 (harvest) months post-seawater transfer. *L *lordosis, *K *kyphosis, *S *scoliosis. Values in brackets represent numbers of affected individuals. n = 284.

Month	n (rSC)	L-only	K-only	S-only	L + K	L + S	K + S	L + K + S
1	11	36.4% (4)	45.5% (5)	18.2% (2)	0.0% (0)	0.0% (0)	0.0% (0)	0.0% (0)
2	1	0.0% (0)	100.0% (1)	0.0% (0)	0.0% (0)	0.0% (0)	0.0% (0)	0.0% (0)
4	2	100.0% (2)	0.0% (0)	0.0% (0)	0.0% (0)	0.0% (0)	0.0% (0)	0.0% (0)
5	4	25.0% (1)	75.0% (3)	0.0% (0)	0.0% (0)	0.0% (0)	0.0% (0)	0.0% (0)
8	25	44.0% (11)	36.0% (9)	0.0% (0)	20.0% (5)	0.0% (0)	0.0% (0)	0.0% (0)
11	112	17.9% (20)	19.6% (22)	3.6% (4)	29.5% (33)	0.0% (0)	5.4% (6)	24.1% (27)
13	129	11.6% (15)	16.3% (21)	0.0% (0)	13.2% (17)	0.0% (0)	4.7% (6)	54.3% (70)
Total	284	18.7% (53)	21.5% (61)	2.1% (6)	19.4% (55)	0.0% (0)	4.2% (12)	34.2% (97)

#### Relationships between curvature types and spinal regions

While the average number of affected regions increased with time, individuals predominantly had curvature in either one (29.2%) or all four (28.9%) spinal regions. Where individuals had curvature in two (21.8%) or three (20.1%) regions, affected regions were generally adjacent. Region (R)2 was the most frequently affected by spinal curvature (Table 3). Average curvature severity was also highest in this region, followed by R3. R1 and R4 were of approximately equal severity. There were significant associations between particular curvature types and spinal regions (χ^2^_ΜΗ_ = 122.42, p < 0.01): lordosis most frequently occurred in R1 and R3, kyphosis in R2 and R4 and scoliosis in R2 and R3 (Table 3).

**Table 3:** Prevalence and severity of spinal curvature (L = lordosis, K = kyphosis, S = scoliosis, All = all curvature types) in spinal regions R1 (V1-V8), R2 (V9-V31), R3 (V32-V50) and R4 (V51-V62 +) of Study I individuals. Values in brackets represent numbers of affected individuals. n = 284.

Spinal region	Prevalence				Severity
	All	L	K	S	All
R1	56.7% (161)	67.8% (139)	11.1% (25)	12.2% (14)	0.63 ± 0.60
R2	83.1% (236)	33.7% (69)	88.4% (199)	53.0% (61)	1.11 ± 0.75
R3	53.9% (153)	75.5% (105)	11.6% (26)	81.7% (94)	0.78 ± 0.84
R4	55.3% (157)	15.6% (32)	63.6% (143)	15.7% (18)	0.62 ± 0.61

#### Relationships between curvature types and concurrent vertebral body anomalies

Most individuals with rSC (67.3%, n = 191) possessed curvature alone (SC-only). Vertebral body anomalies (VA) were observed in 93 individuals with curvature (SC + VA) and 295 without (VA-only) (Table 4). The number of individuals with SC + VA at each assessment increased throughout the study, peaking in M13, but was consistently lower than the number of SC-only individuals. Average curvature severity was significantly higher for SC + VA than SC-only individuals (*U* = 5834.50, p < 0.01).

**Table 4:** Prevalence of vertebral body anomalies (VA) in Study I individuals affected (SC + VA) and unaffected (VA-only) by radiographic spinal curvature (SC). F = fusion, C = compression, VS = vertical shift. Values in brackets represent numbers of affected individuals. n = 388.

Group	n	F	C	VS	F-only	C-only	VS-only	F + C	F + VS	C + VS	F + C + VS
SC + VA	93	14.0% (13)	82.8% (77)	73.1% (68)	4.3% (4)	19.4% (18)	10.8% (10)	3.2% (3)	2.2% (2)	55.9% (52)	4.3% (4)
VA-only	295	18.6% (55)	42.7% (126)	65.4% (193)	8.1% (24)	22.0% (65)	42.4% (125)	4.4% (13)	1.0% (3)	17.0%(50)	5.1% (15)

Compressions (C) and vertical shifts (VS) frequently co-occurred and were prevalent in both SC + VA and VA-only groups, but more so in the SC + VA group (Table 4). Fusions (F) were the least prevalent, but more common in VA-only individuals. Concurrent compression and vertical shift (C + VS) was the most prevalent VA combination in the SC + VA group and vertical shift alone (VS-only) in the VA-only group (Table 4). While there were no between-group differences in summed fusion severity (*U* = 14,317.00, p = 0.34), compression and vertical shift severity were significantly higher in the SC + VA group (*U* = 7,375.50 and 10,615.00 respectively, p < 0.01).

In individuals with rSC, VAs most frequently occurred in R3 (46.1%) and least in R1 (15.1%). Equal proportions (35.5%) were observed in R2 and R4. There was a significant association between curvature and VA location (χ^2^ = 17.68, p < 0.05), however further analysis revealed this only applied to compressions (χ^2^ = 26.77, p < 0.05). Co-existence of compressions in the same region as spinal curvature occurred in 41.2% of individuals with C + L, 21.3% with C + K and 61.4% with C + S.

### Study II

#### Mortality

Total mortality of the post-inventory loss population (n = 241) between M5 and M15 was 70.1% (n = 169). Most of this mortality occurred in the M9–M11 period, whereupon 34.4% (n = 83) of the population died. Mortality in the M5–M9, M11–M13 and M13–M15 assessment periods was 15.4% (n = 37), 16.6% (n = 40) and 3.7% (n = 9) respectively.

Over a quarter (26.6%, n = 45) of the early mortalities had radiographic spinal curvature at one or more assessments prior to death. Most of these individuals (68.9%, n = 31) died during the M11–M13 period, with the remaining 14 dying in M5–M9 (n = 3), M9–M11 (n = 6) and M13–M15 (n = 5).

#### Onset, prevalence and severity of spinal curvatures

Twenty-five (39.7%) individuals developed radiographic curvature (rSC) during the study. Curvature first appeared radiographically at M11, when seven individuals were affected (Supplementary Table 3). Five of these individuals had the highest summed curvature severities at harvest. Most of the remaining individuals (n = 13) developed rSC between M11 and 13. Five individuals had curvature at M15 only. At M15, 25 fish had rSC. Curvature was externally visible in 15 of these individuals, all of which had a summed curvature severity of at least 3. Mean curvature severity increased over time, with the greatest increase occurring between M11 and 13. Body weight (BW) at curvature onset ranged between 970–5470 g, averaging 2,666.80 ± 250.42 g. There were no BW differences between individuals affected and unaffected by curvature (Supplementary Table 4). Scale loss, eye anomalies, lesions and fin rot prevalence at M15 were similar between individuals with and without rSC but spots were more prevalent in unaffected individuals (Supplementary Table 5).

#### Relationships between spinal curvature types

The results of Study II were similar to those in Study I. Kyphosis was the most common curvature type and frequently co-occurred with lordosis. At the point of curvature onset, one individual had L-only, five had K-only, 13 had L + K, one had K + S and five had L + K + S. Scoliosis typically developed after lordosis and/or kyphosis and seldom occurred where one or both of these types were absent. S-only and L + S were never observed and only one individual had K + S during the study. By M15, 16 of the 25 individuals with curvature (64.0%) had L + K + S. A further 4 had L + K, 1 had K + S and 4 had K-only.

#### Relationships between curvature types and spinal regions

Given that many individuals possessed curvatures in more than one spinal region at radiographic onset, it was difficult to determine in which region curvatures initially developed (Supplementary Table 6). However, individuals with only one curvature type at onset were almost exclusively affected in R1 or R2. Where only the most severely affected region was scored for each curvature type, the relationships between curvature types and spinal regions were highly similar to those reported in Study I. Lordosis most frequently occurred in R1, R2 and R3, kyphosis was almost exclusively observed in R2 and R4, and scoliosis was only found in R2 and R3.

**Figure 1:**
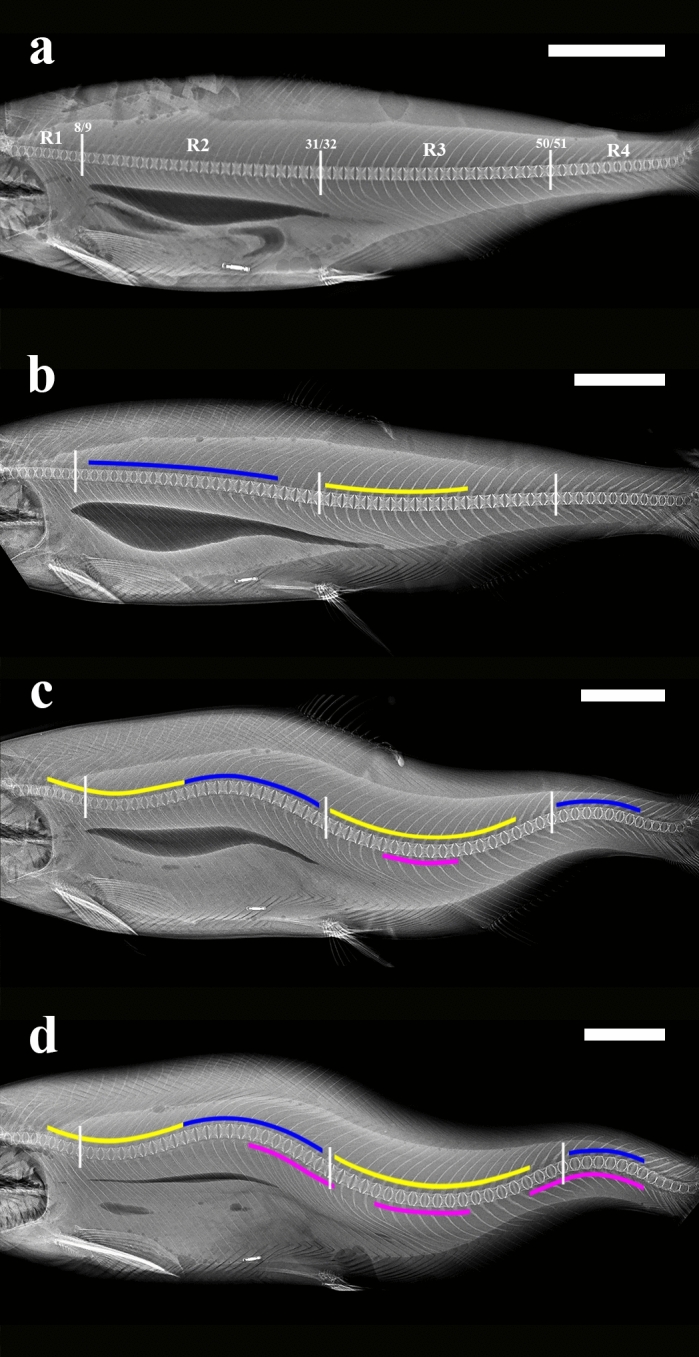
Progression of spinal curvature development evident on lateral radiographs of a Study II individual at assessments 5 (**a**), 11 (**b**), 13 (**c**) and 15 (**d**) months post-seawater transfer. Labels R1, R2, R3 and R4 in (**a**) correspond to spinal regions 1 (vertebra (V)1–8), 2 (V9–31), 3 (V32–50) and 4 (V51–62 +). Small vertical white lines in (**a**)–(**d**) indicate the transition points between each of the four spinal regions. Coloured lines indicate lordosis (yellow), kyphosis (blue) and scoliosis (pink). Scale bars = 5 cm. (**a**) 5 months post-seawater transfer, pre-radiographic curvature onset. All vertebrae exhibit the regular “X in a box shape”, are evenly spaced, and are dorso-ventrally aligned with one another in all regions. The vertebral column exhibits no angular deviation indicative of spinal curvature. (**b**) 11 months post-seawater transfer. Radiographic curvature onset has occurred, with kyphosis present in R2 and lordosis in R3. R1 and R4 are unaffected. (**c**) 13 months post-seawater transfer, post-radiographic curvature onset. Lordosis is now present in R1 as well as R3, and kyphosis in R4 in addition to R2. The angle of curvature of the existing lordosis in R2 and kyphosis in R3 has increased, and scoliosis has developed in R3 at the apex of the lordotic curve in this region. The individual now exhibits a pattern of alternating lordosis and kyphosis down the length of the vertebral column. (**d**) 15 months post-seawater transfer (harvest), end-stage curvature phenotype. The angle of curvature has increased for all existing curvatures, and scoliosis has developed in R2 at the transition zone between the kyphosis in R2 and lordosis in R3, and R4 at the apex of the existing kyphotic curve.

The observations reflect a distinct curvature development pattern which frequently occurred in SC-only individuals in both studies (Fig. 1). Initially, the vertebral bodies and spinal phenotype were indistinguishable from individuals unaffected by curvatures (Fig. 1a). Subsequently, individuals developed lordosis and/or kyphosis in R1 and/or R2 , which was occasionally accompanied by the alternate curvature type in R3 (Fig. 1b). Additional curvatures then manifested in R3 and R4, producing a pattern of alternating lordosis and kyphosis down the length of the spinal column which rapidly increased in severity (Fig. 1c). As severity increased, scoliosis developed, usually in R2 and R3 at the apices of the existing lordotic and kyphotic curves (Fig. 1c,d). In the advanced stages of development, affected individuals possessed multiple lordotic, kyphotic and scoliotic curvatures down the length of the spinal column (Fig. 1d).

#### Relationships between curvature phenotypes and concurrent vertebral body anomalies

Divergence from the typical curvature progression and end-stage spinal phenotype occurred in individuals with SC + VA (Supplementary Fig. 7). Fourteen individuals had SC + VA at M15 (Supplementary Table 8). Average curvature severity of these individuals at harvest (M15) (5.07 ± 0.79) was higher than individuals with SC-only (3.82 ± 0.75) but not significantly (*t* = − 1.13, p = 0.27). VAs arose before curvature onset in 6 individuals, at the same time in 4, and after in 4. As in Study I, C + VS was the most common VA combination and fusions were infrequent.

An additional 13 individuals developed VA but not SC (VA-only). Most possessed compressions and/or vertical shifts, fusions were rare (Supplementary Table 8). VAs were first detected in M5 for 6 fish, M11 for 1, M13 for 4 and M15 for 2. Average VA severity was similar between VA-only and SC + VA groups at both VA onset (*U* = 53.50, p = 0.07) and harvest (*U* = 64.00, p = 0.20).

## Discussion

The results of Study I and II were highly similar in that 43.0% and 39.7% of the respective harvest populations had radiographic spinal curvature. These are higher rates than previously reported for farmed Chinook salmon^23^, but may be the result of handling, as our experimental cohorts were handled more than a typical commercial stock. Though Munday et al*.*^22^ suggested that external trauma was unlikely to cause the peri-vertebral inflammation associated with curvature in farmed New Zealand Chinook salmon, internal trauma and subsequent inflammation caused by handling has been proposed to underpin spinal anomaly development^42^. Additionally, Study I assessments were only conducted on the large grade population after M5. Potentially, individuals in this population grew faster than those which were allocated to the small and mixed grade populations. Fast growth is a widely recognised risk factor for spinal anomaly development^1,19,43^, which may explain why the Study I population exhibited a higher curvature prevalence than previously reported. Although this does not explain the high curvature rate in the Study II population, spinal anomaly prevalence is also known to vary significantly between brood years, farm locations and adjacent pens^1,22^, and may be influenced by differences in environmental conditions (e.g. water temperature, quality and current velocity, dissolved oxygen levels, stocking density, feed composition and amount) during seawater rearing. A number of factors could therefore have driven the curvature rates observed in the current study.

Consistent with previous findings^23^, spinal curvature developed late in production. Indeed, the low rate of curvature we observed in M5 (1.4%, n = 4) was consistent with that reported by Perrott et al*.*^23^ at the same time-point (0.5%, n = 2). Interestingly, prevalence in Study I was significantly higher in M1 than M2, 4 and 5. This may have been due to curvature artefacts in M1 radiographs, which are frequently encountered when scoring radiographic curvature in smolt due to the flexibility of the vertebral column at this life stage (Davie pers. comm.). Alternatively, the difference may have been observed because affected M1 individuals died before M2. Although spinal anomaly-related mortality was < 1% for the M1-M2 assessment period, causes of death were determined by visual examination, which mild spinal anomalies are known to pass through undetected. Prevalence of spinal anomaly-related deaths may therefore have been higher than reported for M1-M2, resulting in the reduced curvature prevalence observed in M2, 4 and 5. Curvature may have also contributed to mortality during the later assessment periods. While spinal anomaly-related deaths accounted for just a small proportion of total mortality in both the pre-grading and post-grading periods of Study I, the prevalence of such deaths was highest during M8–M11 and M11–13, when the increases in curvature prevalence and severity respectively were greatest. Similarly, the greatest increase in curvature severity for Study II was also observed in M11–M13, and constituted the period with the highest mortality rate of individuals with radiographic curvature. In future, radiographing the experimental population prior to commencement of studies, as well as any individuals which die prematurely, will likely help clarify the contribution of curvature to early mortality.

Curvature appeared in significant numbers of fish eight months after seawater transfer (M8) in Study I and eleven months (M11) in Study II. However, prevalence continued to increase until harvest at M13 (Study I) and M15 (Study II), and there was substantial variation in the timing of radiographic onset among individuals within each study. The timing of onset appeared to be associated with curvature severity, with more severely affected individuals developing curvature earlier. This is not an unusual observation as the severity of spinal anomalies, curvature and otherwise, is known to increase with time^12,23^, but why some individuals develop curvature earlier than others warrants further investigation. Average BW of individuals at initial curvature onset was similar between the two studies (Study I = 1882.92 ± 21.37 g in M8 vs. Study II = 1665.87 ± 60.95 g in M11), suggesting that curvature onset may be contingent on reaching or exceeding a particular body weight. However, the BW range of Study II individuals at which spinal curvature was first observed was very broad (970–5470 g). Average BW also only differed between Study I individuals affected and unaffected by curvature at harvest (M13), which is not unusual given that finfish affected by spinal anomalies often have increased metabolic costs post-onset^11–13,44^. Body weight therefore appears unlikely to be responsible for the observed differences in curvature onset. However, as individuals were only weighed a few times over the study period, we cannot exclude the possibility that curvature onset was associated with short periods of accelerated growth between assessments that were not captured. Future studies should therefore evaluate a possible relationship between growth rate and curvature onset, which may explain the observed inter-individual differences.

The current research provides a more refined period of onset for curvature than previously reported^23^, and suggests that the first eight months of the seawater cycle may be particularly critical to the development of curvature. However, our observations also suggest that a clear “window of onset” does not exist. While curvature was rare up to M8, there was no obvious point of onset thereafter; curvature developed at all later stages, including at harvest. Macroscopic radiographic changes are in many cases considered to reflect advanced-stage disease and several weeks can exist between the onset and development of a deformity^19,45,46^. X-ray is also limited in its ability to detect musculoskeletal changes which may precede the onset of radiographic curvature. The initiating events may therefore have occurred, and even resolved, before curvature became radiographically apparent^23^. Indeed, peri-vertebral inflammation has been associated with spinal curvature in Chinook salmon^22^, but this would be undetectable by routine radiography, as would any micro-anatomical changes to vertebral structure. Further investigation using more sensitive, ideally non-invasive, diagnostic tools that can assess both bone and soft tissues [i.e. computed tomography (CT) and magnetic resonance imaging (MRI)] may therefore help determine when the curvature process is initiated. Based on our observations, emphasis should be placed on the first eight months of the seawater cycle, particularly the immediate post-SW transfer (post-smolt) stage, which is considered a critical window for growth and onset of spinal anomalies in salmonids^1,10,47^, as well as any subsequent periods of rapid growth, especially those close to curvature onset.

Strong relationships between the curvature types and the spinal locations in which they developed were observed in both studies. In other species of finfish, each curvature type is typically reported alone (e.g. Grotmol et al*.*^32^, Kranenbarg et al*.*^33^ Chatain^34^, Kihara et al*.*^36^, Yokoyama et al*.*^40,41^) and frequently associated with a particular region of the vertebral column, which can reflect the underlying aetiology^19^. Though lordo-kyphosis, lordo-scoliosis and kypho-scoliosis are occasionally reported^37–39,48^, rarely are all three types documented in the same individual and/or in multiple non-adjacent spinal regions. It is therefore interesting to note that we observed both; a repeating pattern of alternating lordosis and kyphosis occurred from head to tail and was accompanied by scoliosis at the apices and/or junctions between these curvature types, especially in R2 and R3, where most of the anaerobic musculature is deposited. The mechanism by which this curvature condition develops therefore most likely differs from those which produce only one type of curvature in a particular spinal region.

Rather than three co-existing independent anomalies, the three curvature types appear to collectively represent a single condition – a spinal curvature complex. This has been suggested previously for NZ Chinook salmon^23^ and closely resembles the “LSK complex” reported for gilthead seabream^5,26–28,35^, which consists of serial repetition of lordosis, scoliosis and kyphosis from head to tail. Similar conditions have also been reported in Atlantic salmon^15,18^, rainbow trout^49^ and red porgy^50^, but in these cases only the end-stage phenotype was studied. The process by which it develops has not been characterised, likely due to the rarity of these complexes relative to other commercially-important anomalies. Thus, while the end-stage phenotype may be radiographically similar, this does not imply that the processes by which such curvature patterns arise are comparable to those involved in Chinook salmon curvature. In humans, scoliosis has been considered not a single deformity but a curvature complex arising from an initial lordosis, which induces compensatory kyphosis and subsequent twisting of the spinal axis^51^. Given that lordosis and kyphosis in our populations occurred at similar rates and scoliosis manifested shortly after both had developed, it could be speculated that the curvature complex in Chinook salmon arises via a similar process, but this requires further investigation.

In the current study, VAs appeared to have little involvement in curvature development. Most individuals developed curvature alone, and while several individuals with SC + VA exhibited a different end-stage spinal phenotype than those with only curvature, not all individuals with curvature developed VAs and not all individuals with VAs developed curvature. Perrott et al.^23^ reported similar results. While most of their individuals with curvature at harvest had concurrent vertebral compression (46.0%), many did not, indicating that curvature development was not contingent on the presence of VAs. Thus, while where VAs and curvatures co-occur it can be difficult to determine which is the primary anomaly^1,15^, observations from both the current and previous studies suggest that the curvature is the primary malformation in NZ Chinook salmon. VAs therefore probably arise either secondarily or independently, a conclusion supported by the fact that many VAs developed at the curvature apices, which in other species of finfish is considered a response to the altered vertebral mechanical loads produced by spinal curvatures^33,38^.

Overall, the results support previous evidence of a neuromuscular origin for curvature in farmed NZ Chinook salmon. While vertebral body anomalies are considered to arise via skeletogenic processes, deviations of the vertebral column which manifest without vertebral body alterations are thought to have a neuromuscular origin^15^. Indeed, Munday et al*.*^22^ found that the vertebral body structure of harvest-size NZ Chinook salmon with curvature was unchanged from those without, and Lovett et al*.*^21^ found no differences in vertebral mineral content between adult individuals affected and unaffected by curvature. In the current study, many affected individuals developed curvature alone and VAs did not consistently arise prior to curvature onset. Our results therefore contribute to an accumulating body of evidence that neuromuscular rather than bone-related issues are likely responsible for spinal curvature in NZ Chinook salmon.

Although our main aim was to characterise the development of spinal curvature, enough research was performed to speculate about the possible contributing factors. While development of the LSK-complex in gilthead sea bream has been associated with family structure^26–28^, spinal curvature has a low-moderate heritability in NZ Chinook salmon^20,22^. Consequently, although there may be a genetic component to curvature development in NZ Chinook salmon, the predominant causative factors are suspected to be environmental. Swim-bladder malfunction^32–35^, inadequate nutrition^18,50,52^, infectious agents^40,41^, heavy metal toxicity^39^, high rearing temperature^29,30^ and excessive or imbalanced mechanical load due to high intensity/abnormal swimming^34,36–38^ have been linked to spinal curvature in other species of finfish. Curvature onset in many individuals in our study occurred after doubling in BW (average 758.97 g in M5 to 1665.87 in M11), suggesting growth may contribute to curvature development. However, this period of substantial growth overlapped with higher summer water temperatures. Since elevating rearing temperature is a common method of increasing growth rate in farmed finfish, and both high temperatures and fast growth are known drivers of spinal anomalies^1,45,46,53–57^, the extent to which they, either solely or in combination, are risk factors for curvature development warrants further study.

Previous studies have considered curvature in farmed Chinook salmon to have a primarily mechanical aetiology, namely imbalance and/or overload from excessive musculature^21–23^. Indeed, salmon are selectively bred for rapid acquisition of large muscle mass^15^ and farmed Chinook salmon can triple in BW over the last six months of the SW cycle^20^. Deschamps et al*.*^53^ theorised that overload and subsequent deformation of the spinal column could occur where the rate of muscular growth increases without a concomitant increase in vertebral bone deposition*.* Similarly, Kranenbarg et al*.*^33^ reported that lordosis in sea bass may develop due to “buckling” of the spinal column under compressive muscular load during sensitive growth periods, where the mineralized support structures (i.e. vertebrae) are not fully formed. Because a bone-related aetiology for spinal curvature in farmed Chinook salmon cannot be entirely excluded, the biochemistry, structure and integrity of the vertebrae should continue to be evaluated. Where mechanical imbalance is concerned, Hawes and O’Brien^58^ proposed that spinal deformities in humans, notably scoliosis, predominantly arise by sustained asymmetric muscular loading of forces on the spinal column. The pattern of repeating curvatures observed in Chinook salmon in the current study could therefore also be a compensatory response to imbalanced mechanical forces down the length of the spinal column, particularly given that regions R2 and R3, where most of the anaerobic musculature is concentrated and where the greatest mechanical forces are applied to the spinal column^59^, were the most severely affected. Further investigation of the magnitude and distribution of muscular force on the spinal column, as well as muscle and connective tissue (i.e. collagen) integrity, may provide data to evaluate this hypothesis. Indeed, Perrott et al*.*^60^ recently reported differences in the collagen content and cross-link profiles of muscle samples of Chinook salmon collected from farms with high and low rates of spinal curvature, suggesting that continued study of the integrity and condition of soft tissues will likely be important to elucidating the aetiology of spinal curvature.

Interestingly, free-living New Zealand Chinook salmon also develop high rates of spinal curvature^20^, leading to the proposal that the condition may be reflective of the species’ life history, rather than strictly a product of intensive production. Like other Pacific salmonids, Chinook salmon are semelparous and only spawn once in their lifetime^20^, an event which is preceded by sexual maturation and significant physiological changes. In females, these changes are primarily modulated by the hormone 17β-estradiol^61^ and can include a period of rapid “pubertal” somatic growth^20,62^ and increases in muscle fibre power to prepare for spawning migration, followed by cessation of feeding, increases in circulating levels of the stress hormone cortisol^63^, and muscle atrophy^64,65^ as somatic resources are sacrificed to gonad development and vitellogenesis^66^. Reductions in vertebral mineral content due to mobilization of minerals during feeding cessation have also been documented^67,68^. While sexual maturation in farmed New Zealand Chinook salmon is largely suppressed through rearing of exclusively monosex (all-female) populations and photoperiod manipulation after summer solstice, maturation rates of up to 20% in commercial stocks are still reported^61^. Moreover, since the development of secondary sexual characteristics does not generally occur until the later stages of maturation, individuals that appear morphologically immature may in fact be undergoing other associated physiological changes^20^. The possibility therefore exists that physiological changes associated with sexual maturation could contribute to curvature development in farmed New Zealand Chinook salmon.

The present investigation is the first time that spinal curvature development in farmed New Zealand Chinook salmon has been described from onset to end-stage disease. Our results have refined the timeline for radiographic curvature onset and development and provide compelling evidence that there are strong relationships between lordosis, kyphosis and scoliosis and the spinal regions they manifest in. The curvatures therefore do, in fact, collectively constitute a late-onset, rapidly-progressing “LKS” spinal curvature complex. While vertebral body anomalies frequently co-exist with the condition, our findings indicate that the curvatures are the primary anomaly. With a growing body of evidence that the condition is neuromuscular, future studies should implement diagnostic tools which are sensitive to both soft and skeletal tissues, particularly during critical stages of musculoskeletal development, growth, and spinal curvature onset.

## Materials and methods

### Ethics statement

This investigation was approved by The University of Auckland Animal Ethics Committee (protocol 001639). Fish were handled and manipulated in strict accordance with the University’s animal ethics conditions, based on the Animal Welfare Act 1999, and on-farm industry animal welfare regulations.

### Experimental design

#### Study I

In May 2015 (month (M) 0), hatchery-reared female New Zealand Chinook salmon smolt (n = 78,841, age = 89 days post-hatch, mean body weight (BW) = 162.68 g) were transferred to a 6,000 m^3^ commercial sea pen located in Ruakaka Bay, Marlborough Sounds, NZ (41°11′38.121″ S, 174°6′51.74″ E), and reared until June 2016 (M13, 3,733.73 ± 48.03 g). Six months after seawater transfer (M6), fish were graded by size into large, small and mixed weight populations, after which only the large-grade population was studied (N = 25,990, mean BW 1,355.24 g). To minimise disturbance and stress to the experimental population, assessments were carried out in accordance with the timing of routine industry assessments at 1, 2, 4, 5, 8, 11 and 13 months post-seawater (SW) transfer. Each assessment consisted of sampling 300 randomly-selected individuals from the experimental population. Stocking density at each assessment was estimated by the commercial production software Fishtalk Control V4.9.6276.36 (AKVA group, Puerto Montt, Chile https://www.akvagroup.com/software-/fishtalk-control), and ranged between 3.10 and 11.00 kg/m^3^ throughout the experimental period. Mortality was estimated from commercial data obtained from Fishtalk Control between seawater transfer (M0) and harvest (M13), which included the probable cause of death based on visual examination of the carcass.

#### Study II

Prior to seawater transfer, 519 individuals were randomly selected from the Study I population and intra-peritoneally tagged with a Passive Integrated Transponder (PIT) tag (Avid Identification Systems Inc, California, USA), then transferred to a 216 m^3^ sea pen and reared for 15 months. In accordance with the timing of routine industry assessments, all individuals were assessed at 5, 9, 11, 13 and 15 months post-SW transfer. At the first seawater assessment (M5), a significant and unusual inventory discrepancy was observed, whereupon 278 individuals were missing from the population. While a subsequent investigation by the industry revealed no confirmed cause, seal predation was cited as the most likely factor. The study was therefore continued with the reduced population size of 241 individuals. Stocking density at assessments was calculated from total biomass of the population within the pen at assessment divided by pen volume, and ranged between 1.14 and 2.17 kg/m^3^. Mortality for assessments between M5 and M15 was calculated based on the number of individuals present at each assessment. Individuals surviving to harvest (N = 72, mean BW 3,460.28 g) were euthanised by anaesthesia with a 20 ppm solution of AQUI-S (AQUI-S New Zealand Ltd, Lower Hutt, New Zealand)^23,69^ followed by percussion stunning and bleeding.

### Environment and nutrition

Mean daily surface water temperature was 14.6 ± 2.5 °C. Monthly range was 11.3–18.8 °C. The Study I population was fed to satiation using timed automatic feeders on commercial diets (BioMar, Puerto Montt, Chile; Skretting, Hobart, Australia; Ridley, Melbourne, Australia) of 3 mm, 4 mm, 6 mm and 9 mm pellets containing 35–50% protein, 18–32% lipid, 10–50% fishmeal, 7–11% fish oil, 1.0–1.4% phosphorus, and 1000 mg Vitamin C per 100 g of feed. The Study II population was hand-fed to satiation on commercial diets (BioMar, Puerto Montt, Chile; Skretting, Hobart, Australia) of 3 mm, 4 mm, and 9 mm pellets containing 35–50% protein, 18–27% lipid, 36–50% fishmeal, 7–11% fish oil, 1.0–1.4% phosphorus, and 1000 mg Vitamin C per 100 g feed. Fish were fed 10 meals per day in M1, 5 per day up to 250 g BW, 3 per day from 250 g to 1 kg and 2 per day from 1 kg onwards.

### Assessment procedure

At each assessment, individuals from both studies were anaesthetised with a 20 ppm solution of AQUI-S^23,69^, weighed and measured (fork length). Condition factor (K)^70^ was calculated as $100 \times \frac{BW (g)}{{L}^{3}(cm)}$. Each fish was visually examined for scale loss, skin spots and lesions, tail wear, predator damage and eye (i.e. cloudiness), jaw, operculum and spinal anomalies, then laterally radiographed at 60 kV and 0.10 mAs^−1^ using an Atomscope HF80/15 + DLP and Canon CXDI-401C COMPACT receiver plate (image area = 430 × 420 mm, resolution = 3,408 × 3,320 pixels, pixel pitch = 125 µm) set at 50 cm distance. In M9, mechanical issues with the X-ray unit prevented radiographic assessment of Study II fish.

### Radiological grading of spinal anomaly types

**Figure 2:**
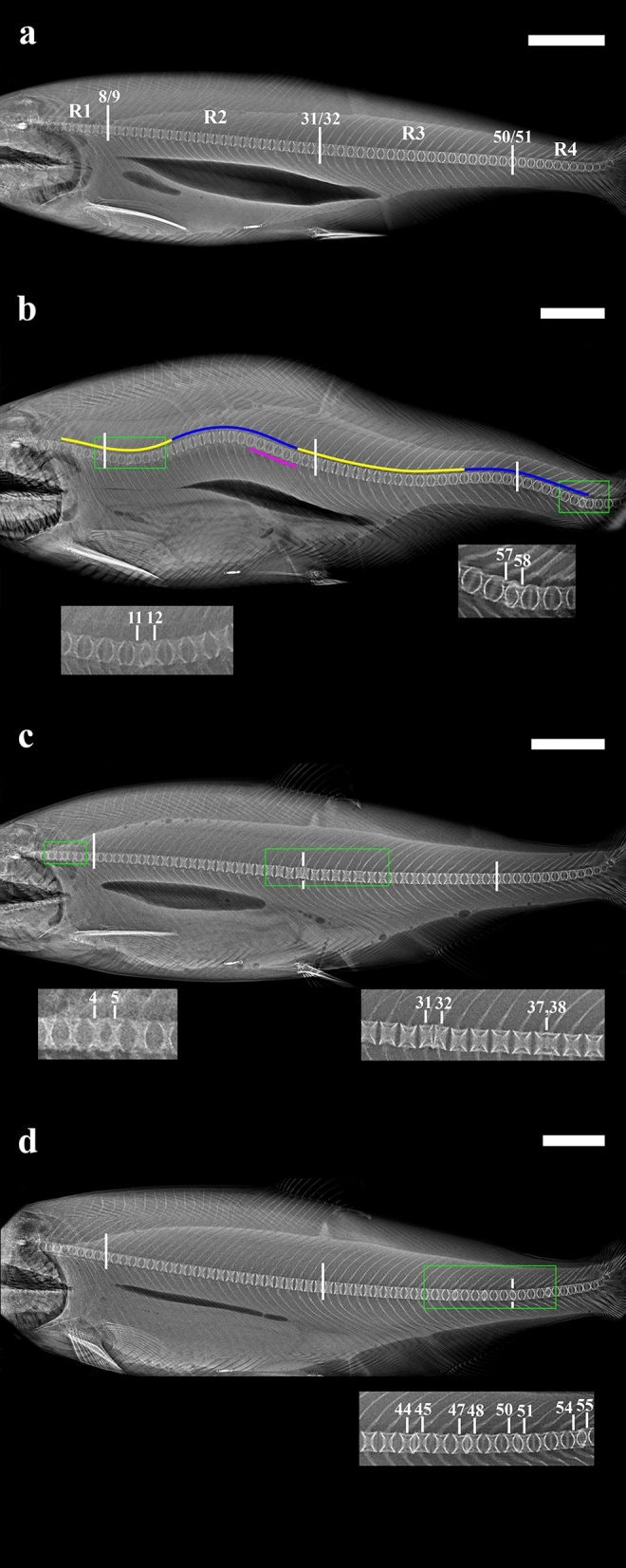
Representative examples of main spinal anomaly types (based on Witten et al*.*^15^) assessed on lateral radiographs of farmed NZ Chinook salmon 13–15 months post-seawater transfer. Labels R1, R2, R3 and R4 in (**a**) correspond to spinal regions 1 (vertebra (V)1–8), 2 (V9–31), 3 (V32–50) and 4 (V51–62+). Small vertical white lines indicate the transition points between each of the four spinal regions. Inset images are included where vertebral body anomalies (vertebral compression, fusion and vertical shift) are present and correspond to the location of green boxes on main images. Numbers in insets represent vertebra numbers. Scale bars = 5 cm. (**a**) No anomaly detected (NAD). All vertebrae exhibit the regular “X in a box shape”, are evenly spaced, and are dorso-ventrally aligned with one another in all regions. The vertebral column exhibits no angular deviation indicative of spinal curvature. (**b**) Spinal curvature (Types 14–16^15^). Lordosis (curvature towards the ventral surface, Type 14^15^), is indicated in yellow, kyphosis (curvature towards the dorsal surface, Type 15^15^) in blue, and presumed scoliosis (lateral curvature discernible on lateral radiographs as change in vertebral shape from “X in a box” to biconcave, Type 15^15^) in pink. Vertebral compression as reduction in intervertebral space (Type 1^15^) and vertical shift (Type 17^15^) is also present at vertebrae (V)11–12 and V57–58. (**c**) Vertebral compression as reduced intervertebral space (Type 1^15^) at V4–5 and V31–32. V31–32 also exhibit one-sided compression (Type 5^15^). V37–38 are almost completely fused (Type 6 progressing to Type 7^15^). (**d**) Vertebral compression as reduction in intervertebral space (Type 1^15^) and vertical shift (Type 17^15^) present at V44–45, V47–48, V50–51 and V54–55.

Spinal anomalies were assessed on lateral radiographs using established protocols^15,22,23^. Each of four spinal regions (Fig. 2a): Region 1 (R1) = vertebra (V) 1–8, Region 2 (R2) = V9–31, Region 3 (R3) = V32–50, Region 4 (R4) = V51–62 +)^68,71^ was assigned a score of between 0 and 3 for spinal curvature and three types of vertebral body anomaly (VA) (Fig. 2b–d), as defined by Witten et al*.*^15^:

1. *Spinal curvature *(*SC*): axial deviation of the vertebral column—lordosis (L), kyphosis (K) and scoliosis (S) (Witten Types 14–16^15^, Fig. 2b). Each curvature type was scored separately. As only lateral X-rays were obtained, diagnosis of scoliosis was presumed based on observation of altered vertebral body appearance, from regular “X in a box” shape to biconcave^15^.
2. *Fusion* (*F*): complete loss of intervertebral space between adjacent vertebrae (Types 6–8^15^) or incomplete/remodelled fusions, cranio-caudal elongation of the centrum with multiple haemal and/or neural processes (Type 7^15^) (Fig. 2c).
3. *Compression *(*C*): reduced intervertebral space, loss of the regular internal vertebral centrum “X” structure and/or one-sided or homogeneous compression of centra (Types 1–5^15^) (Fig. 2c,d).
4. *Vertical Shift* (*VS*): dorso-ventral vertebral body dislocation (Type 17^15^) (Fig. 2d).

Scoring for spinal curvatures was based on angle of deviation from a straight line along the cranio-caudal axis, and for vertebral body fusion, compression and vertical shift on the number of affected vertebrae:

0 = anomaly absent/no vertebrae affected.

1 = mild—1 vertebra affected/curvature angle of 0°–20°

2 = moderate—2–5 vertebrae affected/curvature angle of 20°–40°

3 = severe—> 5 vertebrae affected/curvature angle > 40°

Fish with neither spinal curvature (SC) nor vertebral body anomalies (VA) were referred to as NAD (no anomaly detected), those with only SC or VAs were SC-only and VA-only respectively, and those with both were SC + VA.

### Data and statistical analyses

Adobe Photoshop 2020 V21.1.2 (https://www.adobe.com/nz/products/photoshop) was used to generate the figures and statistical analyses were conducted using R V3.4.3 (https://www.r-project.org) and IBM SPSS Statistics 25 (https://www.ibm.com/products/spss-statistics). Only individuals with complete radiographic and morphometric data were analysed (N_Study I_ = 1,961, N_Study II_ = 63). Visual health data were not available for M1. Predator damage and operculum and jaw anomalies were excluded from the dataset as they were either absent or extremely rare (N < 10). Tail wear was also excluded because almost every fish was affected. Where appropriate, results were reported as means ± standard error (SE). Significance was accepted at p < 0.05.

Chi-squared (χ^2^) tests were used to test for differences in radiographic curvature prevalence between assessments and associations between curvature and VA regions. Associations between the curvature types and their relationships with particular spinal regions were evaluated using Cochran–Mantel–Haenszel (CMH, χ^2^_MH_) tests, a test of association used for conducting multiple Chi-square tests across multiple groups. Non-significant Woolf’s tests confirmed that the assumption of homogeneity of odds ratios required for CMH was met. However, many individuals possessed curvatures of the same type in multiple spinal regions, some of which were adjacent, which violated the assumption of independence of observations within the “spinal region” variable. Consequently, only the region containing the apex (peak) of the most severe curvature for each type was included in the curvature type vs. region analysis for each individual. Relationships between curvature severity and visual curvature prevalence were evaluated using point-biserial correlation (*r*_PB_, a variation of Pearson’s correlation which tests the strength of association between a binary (visual curvature) and a continuous (curvature severity) variable. Independent *t* tests (*t*) were carried out to detect differences in radiographic spinal curvature and vertebral body anomaly severity , as well as in weight (g), length (cm) and condition factor (K), between groups of fish affected and unaffected by spinal curvature. The assumptions of homogeneity of variance and normality for point-biserial correlation and *t* tests were investigated using Levene’s and Shapiro-Wilks tests respectively. Where these assumptions were violated, non-parametric Mann–Whitney *U* tests (normality) and *t* and p values adjusted for heterogeneity were used.

## Supplementary information

Supplementary Information.

## Data Availability

The datasets generated during and/or analysed during the current study are not publicly available due to industry restrictions, but may be obtained from the corresponding author on reasonable request with permission of The New Zealand King Salmon Company Ltd.
