# Supplementary material for: Radiographic characterisation of spinal curvature development in farmed New Zealand Chinook salmon Oncorhynchus tshawytscha throughout seawater production
Source: Sci Rep. 2020 Nov 18;10:20039. doi: 10.1038/s41598-020-77121-y (PMC7674505; doi:10.1038/s41598-020-77121-y)
Supplement: Supplementary file 1 — Supplementary Information. [file 41598_2020_77121_MOESM1_ESM.pdf]

Radiographic characterisation of spinal curvature development in farmed New Zealand Chinook salmon *Oncorhynchus tshawytscha* throughout seawater production

Lovett BA, Firth EC, Tuck ID, Symonds JE, Walker SP, Perrott MR, Davie PS, Munday JS, Preece MA, Herbert NA

Supplementary Table 1. Mean body weight (BW) ± S.E of Study I individuals with (n = 284) and without (n = 1,677) radiographic spinal curvature (rSC) at assessments 1, 2, 4, 5, 8, 11 and 13 (harvest) months (M) post seawater-transfer. Significance values pertain to independent t-tests/non-parametric Mann Whitney U tests of BW differences between groups with and without radiographic spinal curvature at each assessment.* denotes statistically significant differences between groups. † = mean and standard error undefined as only one individual was diagnosed with curvature at this assessment.

**Assessment**	**Mean BW (g)**			**Significance**
	**All**	**No rSC**	**rSC**	**(*t/U*, p)**
**M1**	163 ± 1.3	162 ± 1	169 ± 5	*U* = 1270.00, 0.29
**M2**	390 ± 7	390 ± 7	320†	*t* = 0.62, 0.54
**M4**	679 ± 9	679 ± 9	658 ± 35	*U* = 253.00, 0.78
**M5**	953 ± 13	955 ± 14	779 ± 133	*U* = 382.00, 0.24
**M8**	1883 ± 21	1884 ± 22	1870 ± 72	*t* = 0.19, 0.85
**M11**	2743 ± 34	2766 ± 45	2704 ± 49	*t* = 0.88, 0.38
**M13**	3734 ± 48	3851 ± 67	3578 ± 66	*U* = 8344.50, < 0.01*

Supplementary Table 2. Prevalence of external aberrations in Study I individuals with and without radiographic spinal curvature (rSC) at assessments 1, 2, 4, 5, 8, 11 and 13 (harvest) months (M) post-seawater transfer. n = 1,756.

**Assessment**	**n**			**Scale loss**			**Eye Anomalies**			**Spots**			**Lesions**			**Fin split/rot**		
	**All**	**No rSC**	**rSC**	**All**	**No rSC**	**rSC**	**All**	**No rSC**	**rSC**	**All**	**No rSC**	**rSC**	**All**	**No rSC**	**rSC**	**All**	**No rSC**	**rSC**
**M1**	205	194	11	-	-	-	-	-	-	-	-	-	-	-	-	-	-	-
**M2**	294	293	1	50.3% (148)	50.5% (148)	0% (0)	65.0% (191)	64.9% (190)	100% (1)	1.4% (4)	1.4% (4)	0% (0)	0.3% (1)	0.3% (1)	0% (0)	1.0% (3)	1.0% (3)	0% (0)
**M4**	288	286	2	63.9% (184)	64.0% (183)	50.0% (1)	90.6% (261)	90.9% (260)	50.0% (1)	6.3% (18)	6.3% (18)	0% (0)	0.7% (2)	0.7% (2)	0% (0)	1.0% (3)	1.1% (3)	0% (0)
**M5**	294	290	4	71.4% (210)	71.7% (208)	50.0% (2)	40.1% (118)	40.0% (116)	50.0% (2)	27.6% (81)	27.2% (79)	50.0% (2)	8.5% (25)	8.6% (25)	0% (0)	0.7% (2)	0.7% (2)	0.0% (0)
**M8**	277	252	25	67.2% (186)	66.7% (168)	72.0% (18)	64.3% (178)	63.1% (159)	36.0% (9)	42.2% (117)	42.9% (108)	36.0% (9)	49.8% (138)	48.4% (122)	64.0% (16)	10.8% (30)	11.5% (29)	4.0% (1)
**M11**	303	191	112	37.3% (113)	36.7% (70)	38.9% (43)	8.9% (27)	10.5% (20)	6.3% (7)	59.7% (181)	59.2% (113)	60.7% (68)	19.8% (60)	18.3% (35)	22.3% (25)	13.9% (42)	13.6% (26)	14.3% (16)
**M13**	300	171	129	46.0% (138)	48.0% (82)	43.4% (56)	0% (0)	0% (0)	0% (0)	56.3% (169)	59.2% (95)	57.4% (74)	33.7% (101)	33.9% (58)	33.3% (43)	17.3% (52)	19.3% (33)	14.7% (19)
**Total**	**1,756**	**1,483**	**273**	**55.8% (979)**	**57.9% (859)**	**44.0% (120)**	**47.2% (828)**	**52.3% (775)**	**19.4% (53)**	**32.6% (570)**	**28.1% (417)**	**56.0% (153)**	**18.6% (327)**	**16.4% (243)**	**30.8% (84)**	**7.5% (132)**	**6.5% (96)**	**13.2% (36)**

Supplementary Table 3. Onset (initial radiographic detection), prevalence and severity of radiographic spinal curvature (rSC) and presence of visual SC (P = present, A = absent) in Study II individuals with radiographic curvature at harvest at assessments 5, 11, 13 and 15 (harvest) months (M) post-seawater transfer, in order of increasing harvest curvature severity. n = 25. BW = body weight (g). L = lordosis, K = kyphosis, S = scoliosis. Coloured cells indicate rSC onset for each individual. Dashes indicate the absence of a given anomaly.

Fish ID	Visual SC M15	BW (g) at rSC onset	rSC sev M5	rSC sev M11	rSC sev M13	rSC sev M15	rSC types M11	rSC types M13	rSC types M15
**15616039**	A	4890	0	0	0	1	-	-	K
**42610872**	A	3080	0	0	1	1	-	K	K
**73593256**	A	3280	0	0	3	1	-	L+K	L+K
**0A00065617**	A	3750	0	0	0	1	-	-	K
**14260345**	A	1720	0	0	1	2	-	K	K
**29885009**	A	2050	0	0	2	2	-	L+K	L+K
**42609823**	A	3920	0	0	1	2	-	L	L+K
**95815277**	A	5470	0	0	0	2	-	-	L+K
**107037017**	A	4960	0	0	0	2	-	-	K+S
**107074117**	P	2300	0	0	3	3	-	L+K	L+K+S
**42771042**	A	1170	0	0	4	4	-	L+K	L+K+S
**107032257**	P	1320	0	3	4	4	L+K	L+K+S	L+K+S
**21342850**	P	2570	0	0	3	5	-	L+K	L+K+S
**42629834**	P	2200	0	3	5	5	L+K	L+K+S	L+K+S
**107070031**	P	1490	0	0	3	5	-	L+K	L+K+S
**107070534**	P	1130	0	0	2	5	-	L+K	L+K+S
**0A00143331**	P	2810	0	0	6	5	-	L+K+S	L+K+S
**0A00157114**	P	3410	0	0	0	6	-	-	L+K+S
**42636326**	P	3030	0	0	4	7	-	L+K	L+K+S
**0A00151003**	P	3350	0	0	5	7	-	L+K+S	L+K+S
**42775544**	P	2620	0	3	6	8	L+K+S	L+K+S	L+K+S
**0A00066055**	P	1800	0	1	7	8	K	L+K+S	L+K+S
**42603784**	P	1440	0	2	7	9	L+K	L+K+S	L+K+S
**42605638**	P	970	0	7	7	9	L+K+S	L+K+S	L+K+S
**42783519**	P	1940	0	4	8	9	L+K	L+K+S	L+K+S

Supplementary Table 4. Mean body weight (BW) ± S.E of Study II individuals with (n = 25) and without (n = 38) radiographic spinal curvature (rSC) at harvest at assessments 5, 9, 11, 13 and 15 (harvest) months (M) post-seawater transfer. Significance values pertain to independent t-tests/non-parametric Mann Whitney U tests of BW differences between groups with and without rSC at each assessment. * denotes statistically significant differences between groups.

**Assessment**	**Mean BW (g)**			**Significance (*t/U,* p)**
	**All**	**No rSC**	**rSC**	
**M5**	759 ± 15	769 ± 20	744 ± 24	*U* = 379.00, 0.18
**M9**	1648 ± 39	1630 ± 51	1675 ± 61	*t* = -0.57, 0.57
**M11**	1666 ± 61	1617 ± 78	1741 ± 99	*t* = -1.00, 0.32
**M13**	2464 ± 112	2384 ± 150	2587 ± 168	*t* = -0.89, 0.38
**M15**	3451 ± 195	3375 ± 263	3566 ± 289	*U* = 498.00, 0.75

Supplementary Table 5. Prevalence of external aberrations in Study II individuals with (n = 25) and without (n = 38) radiographic spinal curvature (rSC) at harvest at assessments 5, 9, 11, 13 and 15 (harvest) months (M) post-seawater transfer.

Assessment	n			Scale loss			Eye anomalies			Spots			Lesions			Fin split/rot		
	All	No rSC	rSC	All	No rSC	rSC	All	No rSC	rSC	All	No rSC	rSC	All	No rSC	rSC	All	No rSC	rSC
M5	63	38	25	7.9% (5)	5.3% (2)	12.0% (3)	6.4% (4)	5.3% (2)	8.0% (2)	3.2% (2)	5.3% (2)	0% (0)	1.6% (1)	2.6% (1)	0% (0)	0% (0)	0% (0)	0% (0)
M9				28.6% (18)	26.3% (10)	32.0% (8)	0% (0)	0% (0)	0% (0)	73.0% (46)	86.8% (33)	52.0% (13)	17.5% (11)	13.2% (5)	24.0% (6)	0% (0)	0% (0)	0% (0)
M11				49.2% (31)	50.0% (19)	48.0% (12)	7.9% (5)	5.3% (2)	12.0% (3)	71.4% (45)	76.3% (29)	64.0% (16)	14.3% (9)	7.9% (3)	24.0% (6)	4.8% (3)	5.3% (2)	4.0% (1)
M13				39.7% (25)	42.1 (16)	36.0% (9)	9.5% (6)	0% (0)	24.0% (6)	33.3% (21)	34.2% (13)	32.0% (8)	36.5% (23)	31.6% (12)	44.0% (11)	3.2% (2)	2.6% (1)	4.0% (1)
M15				44.4% (28)	44.7% (17)	44.0% (11)	1.6% (1)	0% (0)	4.0% (1)	22.2% (14)	29.0% (11)	12.0% (3)	25.4% (16)	26.3% (10)	24.0% (6)	4.8% (3)	5.3% (2)	4.0% (1)

Supplementary Table 6. Patterns of radiographic spinal curvature (L = lordosis, K = kyphosis, S = scoliosis) in vertebral column regions 1 (R1, vertebra (V)1-8), 2 (R2, V9-31), 3 (R3, V32-50), and 4 (R4, V50 – 62+) of Study II individuals at initial radiographic detection (onset) and harvest (15 months (M) post-seawater transfer). n = 25. Apex corresponds to the spinal region(s) which contained the peak(s) of each curvature type. Max severity (Max sev) corresponds to the vertebral column region with the highest severity score for each curvature type.

Fish #ID	Regions affected at SC onset	Regions affected at M15	Apex L M15	Apex K M15	Apex S M15	Max sev L M15	Max sev K M15	Max sev S M15
**15616039**	R1	R1	-	R1	-	-	R1	-
**42610872**	R2	R2	-	R2	-	-	R2	-
**73593256**	R1,R2,R3	R4	R4	-	-	R4	-	-
**0A00065617**	R2	R2	-	R2	-	-	R2	-
**14260345**	R2	R1,R2	-	R2	-	-	R2	-
**29885009**	R1,R2	R1,R2	R1	R2	-	R1	R2	-
**42609823**	R1	R1,R2	R1	R2	-	R1	R2	-
**95815277**	R1,R2	R1,R2	R1	R2	-	R1	R2	-
**107037017**	R2,R3	R2,R3	-	R2	R3	-	R2	R3
**107074117**	R2,R3,R4	R3,R4	R3	R4	-	R3	R4	-
**42771042**	ALL	ALL	R2	R2	R3	R2	R2	R3
**107032257**	R2,R3,R4	R3,R4	R3	R2	R3	R3	R2	R3
**21342850**	R2,R3,R4	ALL	R1,R3	R2,R4	R3	R3	R2	R3
**42629834**	R2,R3,R4	ALL	R1,R3	R2,R4	R3	R1	R4	R3
**107070031**	R2,R3,R4	R2,R3,R4	R3	R2,R4	R3	R3	R4	R3
**107070534**	R1,R2	ALL	R2	R1,R2	R3,R4	R2	R2	R3
**0A00143331**	ALL	R2,R3,R4	R3	R2,R4	R2,R3	R3	R2	R3
**0A00157114**	ALL	ALL	R2,R3	R2,R4	R2,R3	R2	R2	R2
**42636326**	ALL	ALL	R1,R3	R2,R4	R3	R3	R4	R3
**0A00151003**	ALL	ALL	R1,R3	R2,R4	R3	R3	R4	R3
**42775544**	R1,R2,R3	ALL	R3,R4	R2,R4	R1,R3	R3	R4	R3
**0A00066055**	R4	ALL	R2,R3	R2,R4	R1,R3	R2	R2	R3
**42603784**	R2,R3	ALL	R2,R3	R2,R4	R3	R3	R2	R3
**42605638**	ALL	ALL	R2,R3	R2,R3,R4	R2,R3	R2	R4	R3
**42783519**	ALL	ALL	R2,R3	R2,R4	R2,R3	R2	R2	R2

Supplementary Figure 7. Variations in end-stage spinal phenotype evident on lateral radiographs of Study II individuals with both spinal curvature (SC) and vertebral body anomalies (VA) at harvest (15 months post-seawater transfer), in order of increasing summed radiographic SC severity. Labels R1, R2, R3 and R4 in (a) correspond to vertebral column regions 1 (vertebra (V)1-8), 2 (V9-31), 3 (V32-50) and 4 (V51-62+). Small white vertical lines in (a) – (f) indicate the transition points between the four vertebral column regions. Coloured lines indicate lordosis (yellow), kyphosis (blue) and scoliosis (pink). Scale bars = 5 cm. (a) Summed curvature severity = 2. Lordosis and scoliosis are present in R3 and kyphosis at the R3/R4 junction. V11 and V12 are completely fused. V10, V13 and V40-42 are compressed. V41 and V42 are vertically shifted. (b) Summed curvature severity = 5. Lordosis is present at the R1/R2 junction and in R3, kyphosis in R2 and at the R3/R4 junction, and scoliosis in R3. V25-28 are compressed, and V26 and V27 are also vertically shifted. (c) Summed curvature severity = 5. Lordosis and scoliosis are present at the R2/R3 junction. Kyphosis is present in R2 and at the R3/R4 junction. V32-35 are compressed and V33 and V34 are also vertically shifted. (d) Summed curvature severity = 7. Lordosis is present at the R1/R2 junction and in R3, kyphosis in R2 and at the R3/R4 junction, and scoliosis at the R2/R3 junction. V40-41 are vertically shifted. (e) Summed curvature severity = 9. Lordosis is present at the R2/R3 junction and in R3, kyphosis in R3 and at the R3/R4 junction, and scoliosis at the R2/R3 and R3/R4 junctions. V29-31 and V44-46 are compressed and V30 and V45 are vertically shifted. (f) Summed curvature severity = 9. Lordosis is present at the R1/R2 junction and in R3, kyphosis in R2 and at the R3/R4 junction, and scoliosis in R2. V11-12 and V57-58 are compressed and vertically shifted.

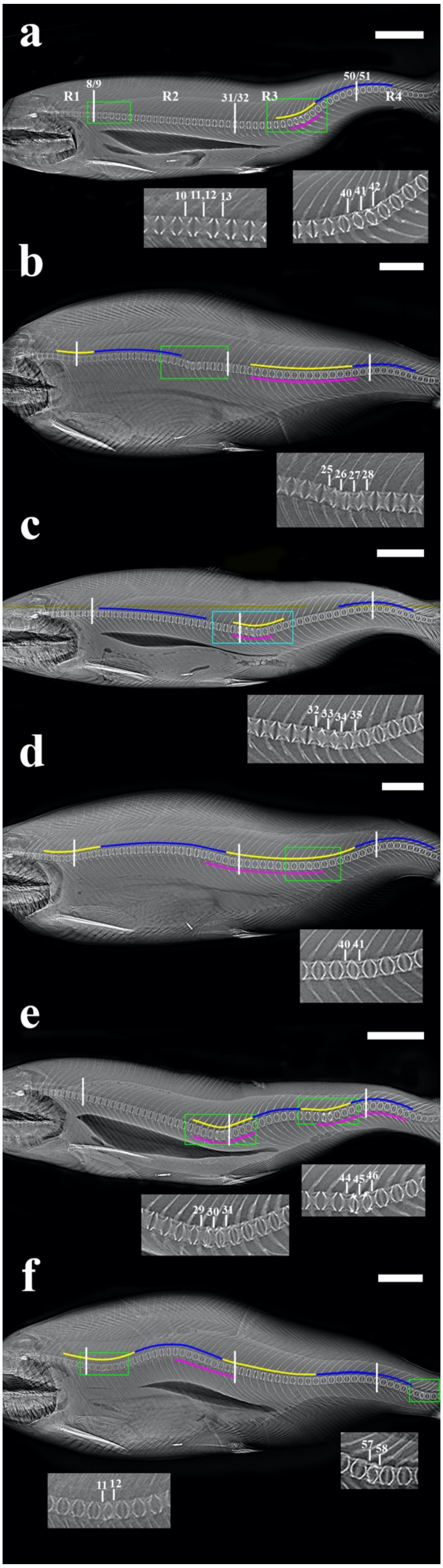
a
8/9
R1
R2
31/32
R3
50/51
R4
10 11,12 13
40 41 42
b
25 26 27 28
c
32 33 34 35
d
40 41
e
44 45 46
29 30 31
f
57 58
11 12

Supplementary Table 8. Onset (initial radiographic detection), prevalence and severity of vertebral body anomalies (VA) at assessments 5, 11, 13 and 15 (harvest) months (M) post-seawater transfer in Study II individuals affected (SC + VA, n = 14) and unaffected (VA-only, n = 13) by radiographic spinal curvature (rSC). F = fusion, C = compression and VS = vertical shift. P = present, A = absent. n = 27. Dashes indicate the absence of a given anomaly.

Fish ID	Group	rSC onset	VA onset	VA sev M5	VA sev M11	VA sev M13	VA sev M15	F	F onset	F sev onset	F sev M15	C	C onset	C sev onset	C sev M15	VS	VS onset	VS sev onset	VS sev M15
**15616039**	SC + VA	M15	M5	3	2	2	4	P	M5	1	1	P	M5	2	1	P	M15	2	2
**42610872**	SC + VA	M13	M5	4	4	6	6	P	M5	2	2	P	M5	2	3	P	M13	1	1
**73593256**	SC + VA	M13	M5	1	1	0	2	P	M5	1	1	P	M15	1	1	P	M15	1	1
**107037017**	SC + VA	M15	M15	0	0	8	8	A	-	-	-	P	M13	4	4	P	M13	4	4
**107032257**	SC + VA	M11	M11	4	4	8	8	P	M5	2	2	P	M5	2	4	P	M13	2	2
**42629834**	SC + VA	M11	M13	0	0	2	4	A	-	-	-	P	M13	2	2	P	M15	2	2
**107070031**	SC + VA	M13	M13	0	0	4	4	A	-	-	-	P	M13	2	2	P	M13	2	2
**107070534**	SC + VA	M13	M5	5	5	4	3	A	-	-	-	P	M5	3	2	P	M5	2	1
**0A00157114**	SC + VA	M15	M11	0	2	2	2	P	M15	2	2	P	M11	2	0	A	-	-	-
**42636326**	SC + VA	M13	M15	0	0	0	4	A	-	-	-	P	M15	2	2	P	M15	2	2
**42775544**	SC + VA	M11	M5	1	1	5	3	A	-	-	-	P	M13	2	1	P	M5	1	2
**0A00066055**	SC + VA	M11	M15	0	0	0	2	A	-	-	-	P	M15	1	1	P	M15	1	1
**42605638**	SC + VA	M11	M11	0	4	7	4	A	-	-	-	P	M11	2	2	P	M11	2	2
**42783519**	SC + VA	M11	M13	0	0	8	8	A	-	-	-	P	M13	4	4	P	M13	4	4
**14127533**	VA-only	-	M13	0	0	2	4	A	-	-	-	P	M13	1	2	P	M13	1	2
**14256529**	VA-only	-	M5	1	2	1	4	P	M11	2	1	P	M5	1	2	P	M15	1	1
**42619571**	VA-only	-	M15	0	0	0	1	A	-	-	-	P	M15	1	1	A	-	-	-
**42632873**	VA-only	-	M11	0	1	0	1	A	-	-	-	P	M11	1	1	A	-	-	-
**42634351**	VA-only	-	M5	3	1	0	2	A	-	-	-	P	M5	2	1	P	M5	1	1
**42773088**	VA-only	-	M13	0	0	2	1	A	-	-	-	P	M13	2	1	A	-	-	-
**42778094**	VA-only	-	M5	9	9	14	13	P	M5	4	6	P	M5	5	6	P	M5	1	1
**82102311**	VA-only	-	M5	2	4	4	6	P	M11	1	2	P	M5	2	4	A	-	-	-
**91258527**	VA-only	-	M13	0	0	0	2	A	-	-	-	P	M15	1	1	P	M15	1	1
**96576792**	VA-only	-	M13	0	0	0	1	A	-	-	-	A	-	-	-	P	M15	1	1
**107035889**	VA-only	-	M5	1	0	2	4	P	M15	2	2	P	M13	1	1	P	M5	1	1
**107894521**	VA-only	-	M5	14	11	12	10	P	M5	6	3	P	M5	5	5	P	M5	3	2
**0A00151232**	VA-only	-	M15	0	0	0	1	A	-	-	-	A	-	-	-	P	M15	1	1
